# Understanding Aerosol-Mediated Disease Transmission

**DOI:** 10.1021/acscentsci.5c00364

**Published:** 2025-12-04

**Authors:** Abigail C. Dommer, Rommie E. Amaro, Kimberly A. Prather

**Affiliations:** † Groningen Biomolecular Sciences and Biotechnology Institute, 8784 University of Groningen, Groningen 9747 AG, The Netherlands; ‡ Department of Molecular Biology, UC San Diego, La Jolla, California 92093, United States; § Department of Chemistry and Biochemistry, UC San Diego, La Jolla, California 92093, United States; ∥ Scripps Institution of Oceanography, UC San Diego, La Jolla, California 92093, United States

## Abstract

This Outlook aims to update the longstanding treatment of airborne disease transmission through an interdisciplinary lens combining biology, surface chemistry, and aerosol physics, drawing parallels between environmental and human-generated infectious aerosols and examining their effects on human and ecosystem health. By recasting the lung surface as a dynamic interface akin to the ocean surface, this Outlook illustrates the importance of a multidisciplinary approach to elucidate the mechanisms of disease transmission at a depth that enables practical mitigation strategies. The urgency of this analysis is motivated by the evolving nature of airborne pathogens of concern, such as SARS-CoV-2 and influenza, and the global impact of dynamic environments on the poorly understood airborne microbiome.

## Graphical abstract

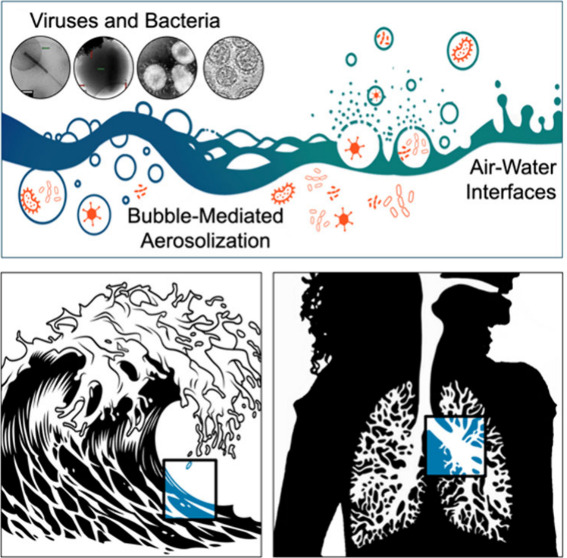

## 1 Introduction

Air is the main pathway for respiratory disease transmission but is often the most overlooked. Air affects the entire global ecosystem, interfacing with all planetary surfaces, whether naturally occurring (e.g., oceans, rivers, soils, and forests) or anthropogenic (e.g., agricultural and industrial waste, exposed sewage, and stormwater runoff). Microbes, biological material, minerals, and pollutants are lofted into the atmosphere and transported between reservoirs, sometimes across entire hemispheres. For example, Saharan dust deposits essential nutrients into the Amazon basin, and at the same time causes red tides along U.S. coastlines.^1^ Fungal spores launched from contaminated soils spread plant disease, and aerosolized bacteria can bring antibiotic resistance to new bacterial communities.^1,2^ Humans in particular exchange 11,000 L of air per day with the environment, and hundreds to thousands of microbes with every breath.^3^ Although most of these microbes in the airborne microbiome are harmless, some are pathogenic and can lead to adverse health effects.^4,5^

In light of the COVID-19 pandemic, as well as revolving outbreaks of measles and avian flu, the airborne spread of disease is an increasing focal point of scientific inquiry.^6^ Persistent threats to global public health include SARS-CoV-2, influenza, respiratory syncytial virus (RSV), and tuberculosis, which are easily spread between individuals through respiratory aerosols (RAs). Unfortunately, the specific mechanisms of respiratory pathogen emission and transfer are not well understood. The factors contributing to pathogen aerosolization and stability may be highly pathogen-specific, complicating not only experimental research but also the implementation of disease mitigation protocols and public safety guidelines.^7–9^ Additionally, efforts to collect and analyze infectious RAs emitted from humans and animal models are often hampered by a suite of experimental limitations.^10,11^

However, pathogen aerosolization is ubiquitous in the natural environment, especially through air/water interfaces like those in our lungs. In this Outlook, we explore how an understanding of the sea-to-air exchange of marine microbes can illuminate our understanding of infectious RA generation and transmission mechanisms, including selective pathogen aerosolization and aerostability. We describe how an interdisciplinary approach to studying airborne pathogen transmission, combining aerosol physics, surface chemistry, and microbiology, can guide the direction of research programs and pave the way for improved public health measures and pandemic readiness.

## 2 The Ocean–Lung Parallel

The sea surface and the lung surface are both highly dynamic interfaces with chemical diversity driven by metabolic activity. In the ocean, metabolic processes are largely associated with microorganisms, particularly planktonic protists, bacteria, and bacteriophages, which concentrate at the air–sea boundary.^12–14^ This interface, called the sea surface microlayer (SML), is a critical component of the marine ecosystem. Varying in thickness up to 1 mm in productive ocean regions, the SML supports vast microbial communities that are responsible for carbon and nutrient cycling as well as gas exchange with the atmosphere. Lung fluid, on the other hand, is secreted by respiratory epithelial cells as products of basal metabolism and in response to stimuli as a part of cell signaling and immunogenic pathways. This respiratory tract lining fluid (RTLF) is a carrier of antimicrobial agents (e.g., mucus and immunomodulating proteins), and functions as a protective physical barrier to foreign material. Its chemical composition is tightly regulated to maintain proper lung function. Varying in thickness from 70 μm in the upper airways to 100 nm in the terminal bronchioles, RTLF traps most debris in the mucociliary layers of the conducting airways and keeps the distal air spaces clear for gas exchange.

The composition of the SML is controlled largely by microbial activity, which responds to spatiotemporal environmental factors such as solar irradiance, temperature, and nutrient availability. The microbial loop, a complex interplay between phytoplankton, bacteria, and viruses, dynamically recycles and transforms organic matter at the SML (Figure 1a). Lipid surfactants are derived from microbial secretions or the breakdown of cellular membranes—either due to senescence or cell lysis—and can include fatty acids,^15^ phospholipids,^16^ cholesterol, and alkanes.^17^ Amino acids, saccharides, and extracellular hydrolytic enzymes such as lipases, peptidases, and glucosidases are also abundant.^18–20^ High molecular weight components of the SML include lipopolysaccharides, humic acids, and transparent exopolymeric particles (TEPs), the latter of which are comprised primarily of polysaccharides secreted by microbes.^21^ As the microbial loop proceeds, surface coverage by TEPs and surfactants peaks with phytoplankton bloom decay, marking the growth of heterotrophic bacteria associating with the gel-like TEPs forming a living biofilm.^22^

**Figure 1:**
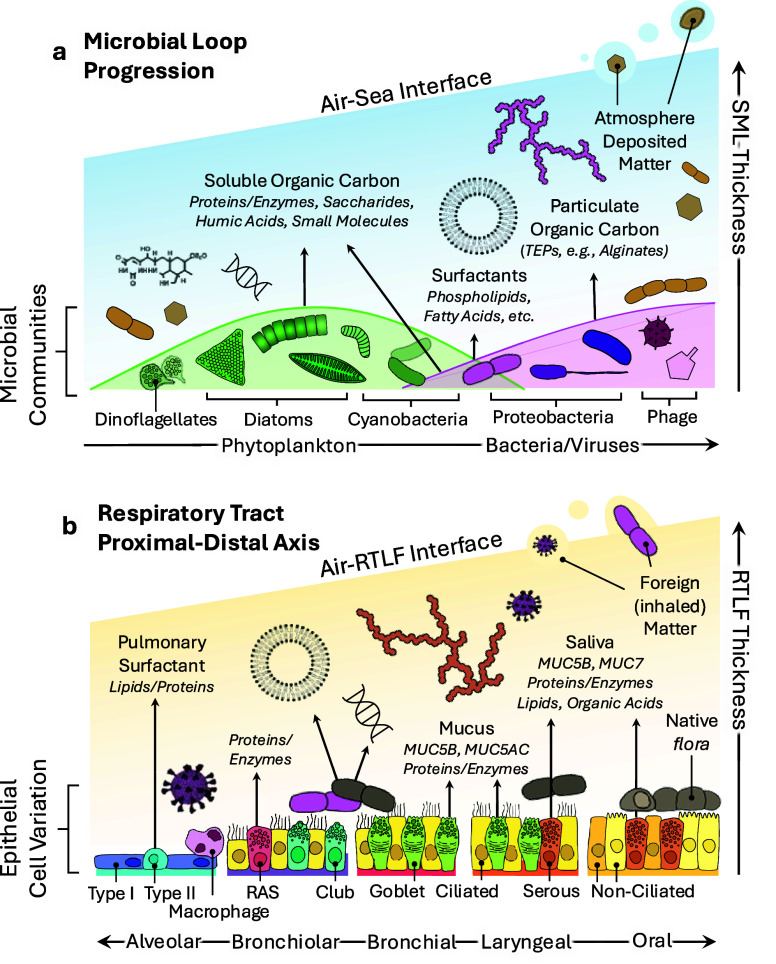
Comparison of metabolic drivers of chemical composition at the a) air/sea interface and b) the air/lung interface. As biological activity increases in the marine environment, the thickness of the organic layer at the interface increases. In comparison, lung fluid thickness increases with increasing mucus production higher in the respiratory tract.

The sea surface and the lung surface exhibit remarkably similar physicochemical properties, playing mirroring ecological and physiological roles in their environments.

In comparison, the biochemical composition of the RTLF varies with cellular differentiation across the proximal-distal axis of the RT (Figure 1b).^23^ In the alveolar epithelia in the distal RT, type-II pneumocytes secrete pulmonary surfactant (PS), composed of PC (>60% DPPC), PG, PI, and PE phospholipids, as well as fatty acids, cholesterol, and surfactant proteins SP-A, SP-B, SP-C, and SP-D, all of which facilitate gas exchange and maintain proper lung function.^24,25^ In the small airways, club cells and respiratory airway secretory cells (RAS) secrete a variety of host defense and anti-inflammatory proteins, including immunoglobulins, collectins, and surfactant proteins SP-A, SP-B, and SP-D.^26,27^ Secretory cells in the upper RT, such as serous cells and goblet cells, are responsible for expressing saliva and mucus, respectively. These fluids owe their characteristic viscoelastic properties in part to mucins, which are high molecular weight linear polypeptides decorated by O-linked glycans. MUC5B and MUC5AC are the predominant gel-forming respiratory mucins, though many others are secreted that participate in the formation of higher order aggregates.^28^ In addition to mucins, saliva contains immunoglobulins, statins, lactoferrin, and a variety of other enzymes, peptides, and lipids.^29^ Respiratory mucus also carries a range of globular proteins, including antiproteases, lysozyme, β-defensins,^30,31^ cytokines, and chemokines.^32^

The SML and RTLF exhibit remarkably similar physicochemical properties (Figure 2) and play mirroring ecological and physiological roles in their environments. Notably, both contain gel-forming macromolecules. Secretion of TEPs in the ocean largely enhances microbial survival;^33^ alginate, for example, produced by brown algae and other microbes, provides structural support to the microbial matrix, traps nutrients, and localizes digestive enzymes.^34,35^ Meanwhile, MUC5B and MUC5AC serve as antimicrobials, where inhaled foreign matter, such as viruses and bacteria, is trapped and removed from the airways by mucociliary clearance. TEPs and mucins both derive their viscosity and elasticity from anionic functional groups, which form noncovalent cross-linked networks using Ca^2+^ and Mg^2+^ bridges. Ca^2+^-chelating organics contribute to a significant enrichment of Ca^2+^ (up to 100-fold) at the SML compared to bulk water.^36–38^ A similar gelation mechanism occurs in the lungs, where Ca^2+^ is bound to mucins during cellular packaging and transport, keeping them condensed into granules. Once in the airway lumen, Ca^2+^ is displaced by Na^+^,^39,40^ triggering mucin expansion and fluidization for efficient clearance. Deficiencies in ion transport channels, such as in cystic fibrosis, can lead to mucin aggregation in the airways, creating hot spots for bacterial colonization.^41^

**Figure 2:**
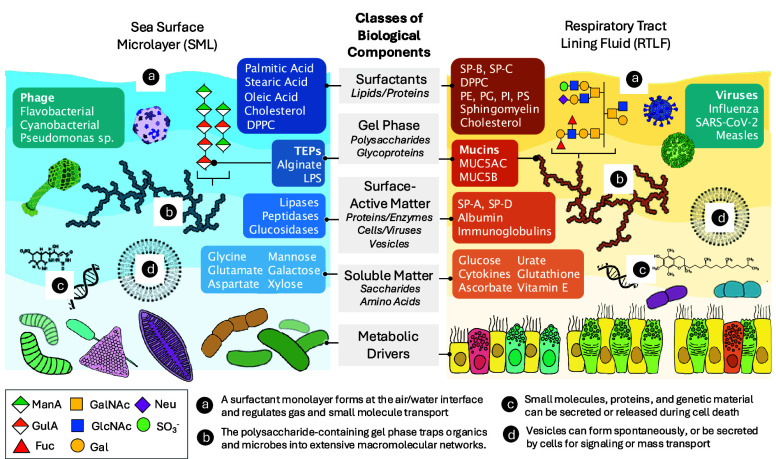
Mirroring chemical compositions of the sea surface microlayer (SML) and the respiratory tract lining fluid (RTLF), and how they serve mirroring ecological and physiological roles in their respective environments. Listed are specific examples of each class of biological component found in a cross section of high thickness, high viscosity regions of the SML and RTLF, and special notes about their structure or function (a-d).

In concentrating matter at the interface, the SML and RTLF also modulate mass transfer, which is critical to both human and whole ecosystem health. The oceans produce at least half of all atmospheric oxygen,^42^ while also serving as a CO_2_ reservoir,^43^ with marine surfactants modulating gas exchange across the interface.^44,45^ In contrast, the composition of PS in the alveoli maintains a low surface tension to ease the work of breathing, allowing the rapid exchange of CO_2_ for O_2_. Further, the exchange of organic matter between phases is inevitable, even across living biofilms. The gel-like, saccharide-rich networks present at productive ocean surfaces and in ciliated airways also concentrate organic matter, which can be aerosolized directly by mechanical agitation. As mentioned above, TEPs effectively trap small molecules, lipids, saccharides, and proteins within their pores. Mucus traps matter by the same mechanisms; in addition to concentrating antimicrobial proteins and peptides, mucins bind a wealth of epithelial cell and lung microbiota secretions, including DNA, polysaccharides, lipid vesicles, and a range of small molecules (Figure 2b–d), many of which have already been identified in RAs.^46,47^ Ultimately, this exchange of biological matter across the interface forms the basis of the airborne microbiome.

Known mechanisms of sea-to-air microbial transfer can enhance our understanding of respiratory pathogen emission.

## 3 Pathogen Aerosolization

Microbial transfer at the ocean-atmosphere interface can provide useful insights into respiratory pathogen aerosolization. Viruses and bacteria that colonize the SML microhabitat are transferred into the atmosphere by bubble bursting as a result of wave breaking or sea foam decay.^48–50^ Sea foam covers up to 6% of the ocean surface,^44,51^ and is common in regions with high surfactant concentrations occurring due to increased biological activity,^44^ microplastic pollution,^52^ or agricultural,^53^ industrial,^54^ and wastewater runoff.^55^ Bubble generation is facilitated by surfactants, which reduce the free energy of bubble formation by lowering the surface tension at the air–water interface. Surfactants also govern foam stability; over time, the aqueous phase drains from the bubble film until destabilizing intermolecular forces cause the film to rupture,^56^ producing particles comprised largely of film components.^57^ Experiments have also shown that the addition of saccharides, proteins, and gels can increase bubble lifetimes by forming extended, cross-linked networks that make up the bubble cap, which slow bubble drainage by increasing aqueous phase viscosity.^58–62^ Critically, however, bacteria and viruses, which have high affinities for surface active material, can also be incorporated into these networks, where they are released into the air upon bubble film rupture.^62–65^

Infectious RAs are produced by film bursting mechanisms in the RT,^66^ and are generally <10 μm in diameter.^67–70^ In the terminal bronchioles, RA generation is driven by small airway reopening.^71^ At the end of an exhale and beginning of an inhale, collapsed airways reinflate, leading to the formation of surfactant-stabilized films akin to bubble film caps. Composed of a thin aqueous bridge coated on either side by surfactant monolayers, each film cap eventually bursts due to incoming airflow, releasing aerosols rich in RTLF (Figure 3).^23,72–74^ In the larynx, RAs are formed by vocal cord vibration, which occurs during phonation activities such as speaking and singing.^75,76^ The vocal folds are also lubricated by RTLF, which stabilizes film bridges and bubble caps that form and burst with vocal fold oscillation.^58,59^ Laryngeal aerosols, rich in viscous respiratory mucins, are distinct in chemical composition from bronchiolar aerosols, which are derived from much thinner fluid of the lower airways.^66^ We note that larger aerosols, produced by turbulent mechanisms in the RT (e.g., sneezing and coughing), more readily settle onto surfaces and are excluded from this survey.^6,77^

**Figure 3:**
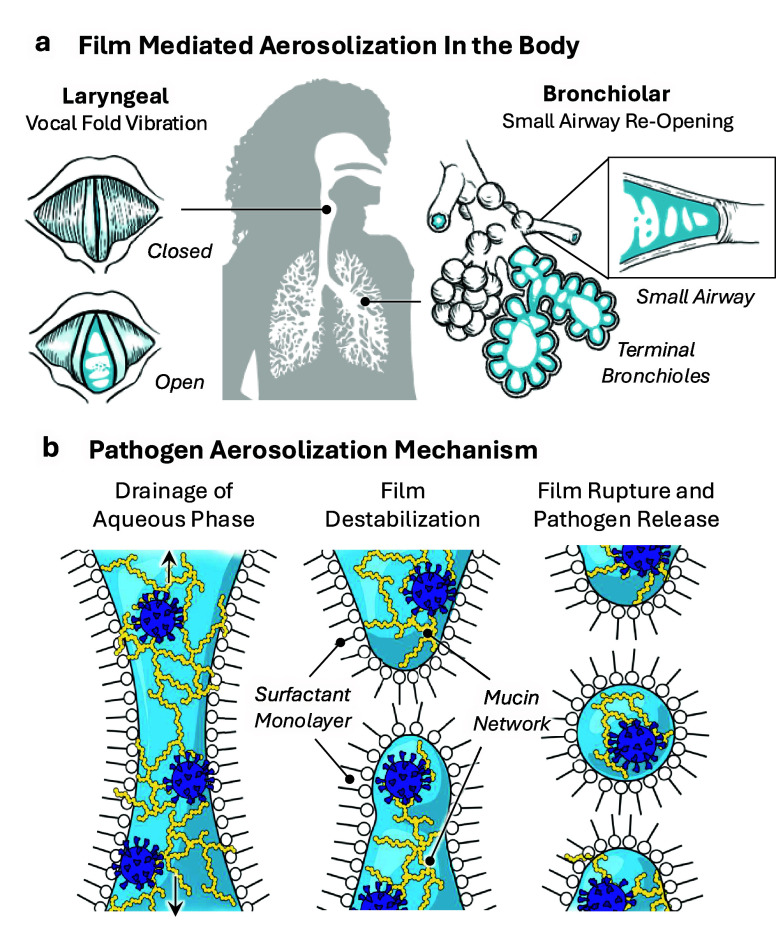
Film-mediated pathogen aerosolization in the body. a) Locations and illustrations of the larynx and bronchioles, which are major sources of infectious respiratory aerosols. b) Mechanism of pathogen (purple) and organic matter (yellow) aerosolization by the bursting of film bridges or filaments.

Although experimental studies are limited, pathogen aerosolization in the airways is likely governed by the same physical principles as the sea-to-air transfer of microbes (Figure 3). A handful of metagenomic studies have characterized the microbes inhabiting sea foam and SML environments, with some beginning to elucidate the mechanisms driving their selective transfer into the atmosphere. Hydrophobicity is one trait frequently, but not exclusively, associated with enhanced microbial aerosolization.^65^ Hydrophobic material adheres to air–water interfaces, and is more likely to be brought to the surface by bubble scavenging. In a laboratory mesocosm study, Michaud et al. observed that bacteria containing mycolic acids on their cell walls (fatty acids with tails up to 90 carbons in length) were enriched in SSA relative to others in their taxa without mycolic acid.^50^ Other studies have found enhanced aerosolization of pigmented bacteria, in particular, those with hydrophobic cell-wall associated pigmentation. Flavobacteria, producing yellow-orange flexirubin-like pigments,^50,78,79^ and some Gammaproteobacteria, producing reddish prodigiosin, were aerosolized more efficiently than less pigmented strains.^80^ Additionally, lipid enveloped viruses (e.g., *Herpesvirales*) were also enriched in the aerosol phase compared to tailed bacteriophages (e.g., *Caudovirales*) which have hydrophilic capsids.^50^ In another study, rising bubbles with a cationic charge were observed to scavenge diatoms and bacteria with negatively charged outer membranes, offering an additional mechanism for microbe transfer.^81^ Applying these enrichment studies to human pathogens can provide useful insights into the nature of respiratory pathogen transmission.

## 4 Airborne Pathogen Stability

In order to survive airborne transport, microbes must withstand a suite of inhospitable conditions, including desiccation, solar irradiation, direct atmosphere exposure, and extremes in temperature, salt, and pH. Some organisms have specifically adapted to survive aerial transport, using wind, for example, for the dispersal of individuals (single cells or spores) or the distribution of genetic material (e.g., pollen). Bacteria such as *E. coli* and *K. pneumoniae* differentially express genes upon aerosolization that enable tolerance to dehydration and starvation.^82–84^ Many bacteria also secrete biofilms, composed of polysaccharide and protein networks akin to TEPs and mucus, which block UV-radiation, inhibit dehydration, and dissipate mechanical damage.^34^ The exine shells of pollen grains are composed of chemically inert sporopollenin,^85,86^ and fungal spores are often protected by highly durable biopolymers such as chitin and glucans. Some spores and pollen grains even undergo morphological changes with decreasing relative humidity, folding up into compact structures optimized to retain moisture and prevent desiccation.^87,88^

For respiratory viruses in particular, which are not metabolically active, growing evidence suggests that coaerosolized organic matter can mitigate the effects of atmospheric stressors by influencing aerosol phase and morphology.^89^ Water loss, controlled by relative humidity (RH), plays a significant role in viral inactivation. Higher RH leads to slower water evaporation, facilitating equilibration with acidic atmospheric gases that can cause chemical disinfection of the virus.^90^ However, the presence of viscous organics, such as respiratory mucins, can also slow the diffusion of small molecules, potentially hindering acidification.^91^ Conversely, under dry conditions, rapid water loss (within seconds), reduces aerosol diameter, increases solute concentrations, and promotes liquid–liquid phase separation (LLPS).^92^ Efflorescence (i.e., crystallization) occurs under a certain RH threshold, and could transiently preserve the virion if it is encapsulated within a salt crystal.^7,89,92^ Many studies have shown that evaporating organic aerosols adopt a heterogeneous semisolid state, where instead of complete LLPS, organic inclusions are interspersed with salt crystals.^93–95^ Oswin et al. observed such mixed-phase heterogeneity in evaporating RAs, with inconsistent measurements of viral infectivity, suggesting that the localization of the virus within the RA may differentially impact its fate.^96^ Thus, the affinity of viruses for particular organics could also be a predictor of aerostability.

pH can also affect viral infectivity, but it can vary in the aerosol phase based on atmospheric processing. Angle et al. observed that nascent SSA pH drops rapidly from that of seawater (pH 8) to <4, with submicron particles reaching pH < 2 within seconds to minutes.^96^ Aerosol size also plays a role in equilibration kinetics, as well as the chemical composition of the aerosol and of the atmosphere. Submicron particles equilibrate with the atmosphere on the order of seconds, while larger particles can take hours to days.^97^ The bicarbonate buffer, present in both RTLF and the ocean, has been of significant interest recently, where bicarbonate evaporation as CO_2_ leads to a spike in aerosol RA pH to >10,^98^ which is then neutralized by atmospheric gases. The kinetics of these processes determine which conditions are important on the time scale of pathogen transmission. For viruses in large particles, e.g., droplets that settle onto surfaces and take a long time to neutralize, tolerance to basic conditions could be important predictors of infectivity.^99^ In contrast, for viruses localized in very small particles, which acidify rapidly and remain airborne for hours, tolerance to acidic conditions may be more relevant.

## 5 Outlook

A robust understanding of the physical properties of the air/sea interface has shaped our knowledge of marine microbial aerosolization. Given the similarities between the SML and the RTLF, we argue that a similar perspective and experimental approach to the lung surface can inform our understanding of RA production and transmission mechanisms.

Perhaps one of the most important unknowns is the chemical composition of RAs. From a top-down approach, RA collection and characterization from human and animal subjects is possible, but biochemical analysis remains a significant hurdle. Precision measurements are hampered by low ambient particle concentrations and high background contamination,^10^ and the size range of RAs spans several orders of magnitude, often requiring multiple sizing instruments operating in tandem. Further, characterizing the sheer chemical complexity of RAs necessitates a broad base of expertise and analytical methods, which is optimized in centers and team-oriented environments. SSAs, like RAs, containing the gamut of macromolecules and whole microbes, have been effectively investigated with center-enabled campaigns. These apply a combination of tools, including mass spectrometry (MS), soft ionization techniques such as extractive electrospray Ionization mass spectrometry (EESI-MS), Aerosol time-of-flight mass spectrometry (ATOFMS), cryo-electron microscopy (cryo-EM), genome sequencing, fluorescence microscopy, and molecular dynamics simulations, among others. Surface and depth sensitive techniques are also critical as compositional gradient can modulate pathogen survival, gas transport and reactions. To date, these methods are often applied individually to RAs,^66^ but a concerted effort, in which samples, methods, and even vocabulary are shared and/or standardized across investigations and disciplines, is urgently needed in ongoing research programs.

Characterizing the sheer chemical complexity of RAs necessitates a broad base of expertise and analytical tools, which are optimized in centers and team-oriented environments.

A bottom-up approach can also be used to investigate RAs, in which model aerosols are generated using surrogate lung fluid and aerosolization techniques. To accurately model nascent RAs, it is imperative to both determine the regiospecific chemical composition of the source lung fluid, and create instrumentation that mimics regiospecific aerosolization mechanisms, many of which are not yet fully understood. The former poses a significant challenge. Generally, RTLF can be collected from targeted lung regions using lavage procedures, although this technique is invasive and unable to isolate bronchiolar from alveolar fluid, underscoring the need for new method development. However, single cell analysis techniques, such as single cell RNA sequencing, has enabled researchers to map the cellular landscape along the proximal-distal axis of the RT, creating cell atlases based on gene expression.^97–99^ Insights from these data sets can guide the preparation of air–liquid interface cultures, from which RTLF can be extracted for chemical analysis.^100^

Secondly, the physicochemical properties of aerosols are sensitive to production mechanism.^101^ Many studies have observed distinctly different particle size distributions and hygroscopic properties between proxy SSAs generated by nebulization versus bubble-mediated production schemes (i.e., glass frits and water impingement).^101,102^ If infectious RAs are produced largely by film bursting mechanisms, it follows that nebulizers, which are commonly used to generate RA proxies, are unlikely to produce particles with the desired properties and behavior. It will be useful to design and test alternative RA generation procedures that more closely mimic physiological processes for laboratory studies. In an important step toward this goal, a few studies have engineered a model larynx for RA production, which approximates vocal fold vibration to produce particles via the bursting of film caps and bridges.^75,103^ In the small airways, aerosol generation proceeds through bubble film-bursting, yet many details of this process remain unclear and are difficult to test. To this end, computational methods are increasingly being used to explore fluid dynamics in conducting airways and alveolar ducts,^74,104–106^ and even toward a virtual human lung.^107^ In close collaboration with experimentalists, existing *in silico* airway models must be extended to evaluate aerosol formation throughout the distal regions.

Finally, insights into mass transfer across the air–liquid boundary can be gained from a molecular-level understanding of the interface. Selective enrichment of both organic and inorganic matter occurs between the bulk, interface, and aerosol phases; while this has been—and continues to be—studied in detail for SSA, it has yet to be investigated for pulmonary interfaces. Combining analytical surface chemistry methods (e.g., Infrared Reflection–Absorption Spectroscopy) and computational methods (e.g., molecular dynamics, MD) can elucidate the mechanisms controlling enrichment across the interface. For example, IRRAS, surface tensiometry, and MD revealed that Ca^2+^ promotes mono- and polysaccharide adsorption to fatty acid monolayers, providing a mechanism for their transfer into SSA.^108^ MD combined with Brewster Angle Microscopy showed that lipases and lipopolysaccharides can adsorb directly to the air/water interface, providing clues to the aerosolization of soluble saccharides and whole proteins.^109^ Similar mechanisms occurring at RTLF interfaces could explain the source of high organic carbon abundances found recently in nascent RAs.^110^

## 6 Conclusions

As environmental conditions continue to shift, the major pathways for airborne disease transmission are being altered, introducing new and unexpected viruses and pathogens into the external environment.^111,112^ Already, the frequency and severity of catastrophic weather events are increasing on an annual basis, driving heavy rainfall and flooding, which in turn increases the aerosolization of waterborne disease.^113–115^ Insufficient infrastructure can lead to sewer and wastewater overflow, exposing local communities to aerosolized sewage, as well as emerging contaminants such as microplastics, biocides, and pharmaceutical chemicals, which can trigger or exacerbate respiratory conditions.^116–120^ Furthermore, inhalation and swallowing of airborne gastroenteric viruses (e.g., norovirus), which are typically waterborne, can cause acute gastrointestinal illness.^118,121^ Indeed, there have been reports of increased gastrointestinal disease in regions experiencing high rainfall and flooding events;^122–124^ coastal communities, already experiencing the effects of rising sea levels, are particularly susceptible.^125^

The tools and insights gained through these studies can be integrated back into the study of the natural environment, in which the airborne microbiome is a persistent threat and is yet a critical part of our ecology.

In this Outlook, we have proposed that the similarities in biochemical composition of RAs and SSAs are traced to the biologically active aqueous interfaces from which they are derived. Mass transfer across the SML occurs according to predictable biophysical properties, which can be extended to form hypotheses about respiratory pathogen emission. Many lessons learned from studying SSAs can guide future RA research, such that existing experimental and computational techniques and infrastructure presently used to study natural aerosol emission and biological systems can be expanded to RA applications. Importantly, team-centered interdisciplinary collaboration is imperative for future research programs. The knowledge gained here can inform public health policy, disease surveillance, risk management, safety protocols and infrastructure development. In turn, these tools and insights can be integrated back into the study of the natural environment, in which the airborne microbiome is a persistent threat, and is yet a critical part of our ecology.

## Supplementary Material



## Notes

Transparent Peer Review report available (PDF)
