# Supplementary material for: Understanding Aerosol-Mediated Disease Transmission
Source: ACS Cent Sci. 2025 Dec 4;11(12):2319–28. doi: 10.1021/acscentsci.5c00364 (PMC12752718; doi:10.1021/acscentsci.5c00364)
Supplement: Supplementary file 1 [file oc5c00364_si_001.pdf]

oc-2025-003644.R1

Name: Peer Review Information for "Understanding Aerosol-Mediated Disease Transmission"

First Round of Reviewer Comments

Reviewer: 1

Comments to the Author

The idea that sea surface microlayer aerobiology is relevant to generation of exhaled breath aerosols has been discussed in scientific fora over the last five years. It is time that these ideas be brought together in a review that can give them a broader audience.

I have two important concerns about the review that can, I think, be addressed with revisions.

First, the description of pulmonary anatomy and physiology is imprecise and, in some places, inaccurate. I've included detailed comments as annotations to the PDF. Some of the generalizations about infectious aerosol size distributions do not, in my opinion, pay sufficient attention to the volume size distribution as opposed to the number distribution. I think that the focus on submicron particles, as opposed to those less than 10 μm is misplaced. This concern arises out of observations influenza virology, as described in the detailed comments. Infectious human influenza virus is filamentous and can be many microns in length, although the diameter is less than 0.2 μm. Influenza viruses may require a high multiplicity of infection to overcome the high frequency of incomplete genome packages. Finally, although influenza virus is present in smaller particles, those less than 1.5 μm do not result in airborne transmission between ferrets. Therefore, I suggest that the review extend the size distribution of interest.

Second, the recommendations for future interdisciplinary research are very good. I the missing point that merits greater emphasis that most models of RTLF use bronchoalveolar lavage data as an indicator of the fluid's composition. However, BAL is a gimish recovered with a saline wash that includes material from large and small airways as well as alveolar spaces. The differences in composition throughout the respiratory tract are important and completely lost. New methods are sorely needed to resolve this knowledge gap.

Reviewer: 2

Comments to the Author

The paper is interesting, however I do not see why the connection between aerosols from the ocean and those from the respiratory tract is important. This argument was not convincing. I learn a lot of interesting details about both, but in the end the only key point that was provided was an assessment that respiratory aerosol studies should use a different aerosolization process other than a nebulizer to better replicate bubble bursting. Is there another key point that I missed? I recommend clearly laying out what was learned from the comparisons made here in this paper. While it is packed with interesting and novel information, what is to be learned from this comparison is not clear enough. Additionally I have only a few minor suggestions to the text.

Page 1

L34: at least in recent history, but in the 1800s it was thought that all diseases were spread through the air, miasma. Perhaps a subtle point, but worth considering that as science has evolved so too has the understanding of disease transmission.

L49: definitely need to add a mention here of measles.

Page 6

Lines 13-14: Do you have a citation for the statement in lines 13-14? About chemical disinfection by atmospheric gases.

Author's Response to Peer Review Comments:

Dear Editors,

Please find our point-by-point response to the reviewers in the attached document.

Best regards,

Rommie

Formatting Needs:

Pull Quotes (Outlook): We encourage you to select 3 - 4 quotes from your Outlook that you would like highlighted in your paper. The quotes should be one sentence-long, unique to the Outlook and not from previously cited work. Please list your quotes at the end of the manuscript file.

Synopsis: ACS Central Science requires a brief synopsis. The synopsis should be no more than 200 characters (including spaces) and should reasonably correlate with the Table of Contents (TOC) graphic. The synopsis is intended to explain the importance of the article to a broader readership across the sciences. Please place your synopsis in the manuscript file after the TOC graphic and label as “Synopsis.”

Reviewer(s)' Comments to Author:

Reviewer: 1

Recommendation: Publish in ACS Central Science after minor revisions noted.

Comments:
The idea that sea surface microlayer aerobiology is relevant to generation of exhaled breath aerosols has been discussed in scientific fora over the last five years. It is time that these ideas

be brought together in a review that can give them a broader audience. I have two important concerns about the review that can, I think, be addressed with revisions.

We are grateful to the reviewer for these comments about the manuscript and agree that it will be great to present the information to a broad audience.

1. Some of the generalizations about infectious aerosol size distributions do not, in my opinion, pay sufficient attention to the volume size distribution as opposed to the number distribution. I think that the focus on submicron particles, as opposed to those less than 10 μm is misplaced. This concern arises out of observations influenza virology, as described in the detailed comments. Infectious human influenza virus is filamentous and can be many microns in length, although the diameter is less than 0.2 μm. Influenza viruses may require a high multiplicity of infection to overcome the high frequency of incomplete genome packages. Finally, although influenza virus is present in smaller particles, those less than 1.5 μm do not result in airborne transmission between ferrets. Therefore, I suggest that the review extend the size distribution of interest.

Thank you for this insight. We have edited the scope of the paper to include aerosol particles with diameters < 10 um.

2. The description of pulmonary anatomy and physiology is imprecise and, in some places, inaccurate. I've included detailed comments as annotations to the PDF.

List of PDF annotations:

- Page 1, Line 45: "Opportunistic" should be deleted. Most exposures to opportunistic pathogens don't lead to health effects because, by definition, they are not pathogenic to immunocompetent hosts. Why focus on opportunistic pathogens and not more concerning pathogenic organisms (e.g. SARS-CoV-2)?
  "Opportunistic airborne pathogen," as defined by Roy and Milton is a different concept and is only a subset of airborne pathogens of concern and not what I think you are trying to get at here.

  We have removed the word "opportunistic" to reduce confusion related to terminology.

- Page 1, Line 49: Human influenza viruses tend to be filamentous and can be as much as 5 μm or more in length. So, it is not entirely clear that human influenza fits the description.

  We have removed the reference to "submicron" here, and have edited the scope of the manuscript to include aerosol particles with diameters < 10 um. (Pg. 4 line 21)

- Page 2, Line 5: Define acronym RA

  RA has been defined.

- Page 2, Line 28: The term "airway" is generally used in pulmonary literature in the sense of "conducting airways" where air is moving on its way somewhere. "Airspace" is more commonly used for areas where there is no bulk flow. Use of "terminal airways" to describe gas exchange regions seems odd. Gas exchange occurs in respiratory bronchioles and alveoli beyond the terminal bronchioles in a region where there is limited or no bulk flow. Hence, it is often referred to as an air space rather than an airway.

  Page 2, Line 28: Debris does reach the alveoli, none the less, and is cleared by alveolar macrophages, unless the debris is toxic to macrophages (silica) or the quantity reaching the alveoli overloads clearance capacity (e.g. coal dust). It might be better to say here that "trapping in the conducting airway mucociliary layers prevents most debris from reaching the gas exchange region of the lung.

  Thank you for this insight! We have revised the sentence to reflect these suggestions:

  > "RTLF thickness varies from 70 µm in the upper airways to 100 nm in the terminal bronchioles, trapping <u>most debris</u> in the mucociliary layers of the <u>conducting airways</u> and keeping the <u>distal air spaces</u> clear for gas exchange"

- Page 2, Lines 36-38: Lung surfactant lipids and proteins are present in the alveoli where they are produced by type-II pneumocytes but are not produced in the conducting airways. Surfactant found in these later areas are carried there by processes that have not been well characterized.

  This brings up a problem with most existing descriptions of the RTLF based on bronchoalveolar lavage. BAL washes out a mixture of moderate and small airways together with alveoli by dilution in saline. It is not possible to tell from this gimish what the RTLF is at any of the individual levels. Nevertheless, there is a paper from AnnaCarin Olin's lab showing that there are surfactant proteins in exhaled breath aerosols.

  Thank you for this comment. We have revised this paragraph to delineate more precisely the regiospecific variations in composition of the RT, rather than presenting the components as a gimish. We specifically report that pulmonary surfactant is produced in the alveoli only and is largely concentrated there.

- Page 2, Line 44: The use of "upper airway" here would be better replaced with "conducting airways." Mucus is produced all the way down to the terminal bronchioles whereas the term upper airways usually means nasopharynx or at least nothing below the mainstem bronchi, rather than everything above the respiratory bronchiole.

  Thank you! This sentence has been revised.

- Figure 1: The implication that the lung microbiome is predominantly in the alveoli is very misleading. The diagram doesn't convey that the lining layer is much thinner in the alveoli. the implication that surfactant proteins are a major constituent of the laryngeal

RLF is not supported by citations and seems unlikely. Ciliated cells should be shown at the bronchiolar level. What is the dotted line above the cells supposed to denote?

Thank you for these comments. We have revised Figure 1 to clarify the regiospecific variations in biological activity with respect to epithelial cell secretions and the lung microbiome. We have also clarified the figure to show explicitly the changing fluid thickness.

- Page 4, Line 13: "Can be" would be more appropriate than "are often." TB is probably the most common infectious aerosol and it is mostly larger than 1 µm. Plus see the next comment about the citations.

Page 4, Line 14 citations 33-36: Only one of these papers shows that the submicron fraction contains culturalble virus; Santarpia 2022. The others included particles as large as 4-5 µm in the fine fraction.

Furthermore, Zhuo et al 2018 found that only aerosol particles >1.5 µm were associated with airborne transmission of influenza between ferrets -- even though virus was present in smaller particles. This might be due to low efficiency of influenza virus such that several virions much reach an individual cell to initiate infection.

Therefore using the term "often" is not well supported. "Can be" submicron would be more appropriate.

Page 4, Lines 23-26: Given that (a) the volume distribution for tidal breathing, and more so for airway closure breathing, is dominated by particles > 1 µm, (b) the residence time for particles in the 1-10 µm range is very significant, and (c) that especially for viruses that have a MOI >1 (see previous comment about influenza transmission in ferrets), it seems problematic to build a review around only particles <1µm. 10µm might be a more suitable cutoff for this review.

We thank the reviewer for these comprehensive notes. We have made the suggested edits, and have expanded the scope of the paper to include aerosols up to 10 um (Pg. 4 line 21)

- Figure 2: The figure suggests that closure is happening in the alveoli or respiratory bronchioles. What evidence is there for closure of alveoli and respiratory ducts (where the concentration of surfactant is very high) rather than at the terminal bronchiolar level and a few generations above that? The figure should be revised to show closure of small ciliated airways. What is shown is a respiratory bronchiole and alveoli -- whereas what should be shown are the last generations of ciliated airways.

We have revised Figure 2 to represent both the bronchioles and the alveoli.

According to this review by Bake *et al.*[1] it still remains unclear exactly where RAs are produced in the small airways. Morawska [2] suggested RAs could be produced in the

respiratory bronchioles, and recent computational models[3,4] of respiratory bronchioles and alveolar ducts, it may also be possible for fluid plugs to form and rupture at these levels.

[1] Bake B, Larsson P, Ljungkvist G, Ljungström E, Olin A-C. Exhaled particles and small airways. Respiratory Research 2019;20:8. https://doi.org/10.1186/s12931-019-0970-9.
[2] Johnson GR, Morawska L. The Mechanism of Breath Aerosol Formation. Https://HomeLiebertpubCom/Jamp 2009. https://doi.org/10.1089/jamp.2008.0720.
[3] He B, Qin C, Chen W, Wen B. Numerical simulation of pulmonary airway reopening by the multiphase lattice Boltzmann method. Computers & Mathematics with Applications 2022;108:196–205. https://doi.org/10.1016/j.camwa.2022.01.013.
[4] Lv Q, He B, Qin C, Wen B. Numerical simulation for pulmonary airway reopening in alveolar duct by lattice Boltzmann method. Computers & Mathematics with Applications 2025;190:206–18. https://doi.org/10.1016/j.camwa.2025.05.005.

- Page 6, Lines 37-38: In the case of viruses from RTLF, however, it is survival in alkaline conditions that may be critical. Note that influenza virus is little affected by alkaline conditions potentially making it better fit for airborne-inhalation transmission than coronaviruses -- a scary thought.

  We thank the reviewer for this insight, and have revised this paragraph to reflect this more accurately.

3. The recommendations for future interdisciplinary research are very good. I the missing point that merits greater emphasis that most models of RTLF use bronchoalveolar lavage data as an indicator of the fluid's composition. However, BAL is a gimish recovered with a saline wash that includes material from large and small airways as well as alveolar spaces. The differences in composition throughout the respiratory tract are important and completely lost. New methods are sorely needed to resolve this knowledge gap.

We have revised the outlook of our manuscript to reflect this insight, adding the following:

> "A bottom-up approach can also be used to investigate RAs, in which model aerosols are generated using surrogate lung fluid and aerosolization techniques. To accurately model nascent RAs, it is imperative to a) determine the regiospecific chemical composition of the source lung fluid, and b) create instrumentation that mimics regiospecific aerosolization mechanisms, many of which are not yet fully understood. The former poses a significant challenge. Generally, RTLF can be collected from targeted lung regions using lavage procedures, although this technique is invasive and unable to isolate bronchiolar from alveolar fluid, underscoring the need for new method development..."

Additional Questions:
Quality of experimental data, technical rigor: Top 5%

Significance to chemistry researchers in this and related fields: Top 5%

Broad interest to other researchers: Top 1%

Novelty: Top 1%

Is this research study suitable for media coverage or a First Reactions (a News & Views piece in the journal)?: Yes

Reviewer: 2

Recommendation: Major revisions required.

Comments:
The paper is interesting, however I do not see why the connection between aerosols from the ocean and those from the respiratory tract is important. This argument was not convincing. I learn a lot of interesting details about both, but in the end the only key point that was provided was an assessment that respiratory aerosol studies should use a different aerosolization process other than a nebulizer to better replicate bubble bursting. Is there another key point that I missed? I recommend clearly laying out what was learned from the comparisons made here in this paper. While it is packed with interesting and novel information, what is to be learned from this comparison is not clear enough. Additionally I have only a few minor suggestions to the text.

We are grateful to the reviewer for this honest appraisal of our manuscript. We have modified Section 4 of our paper from **4:Interdisciplinary Approaches** to **4:Outlook** of the paper to clarify our recommendations. This section begins with the following opening:

“We have highlighted how a robust understanding of the physical properties of the air/sea interface has shaped our knowledge of marine microbial aerosolization. Given the similarities between the SML and the RTLF, we argue that a similar perspective on the RTLF interface can inform our understanding of RA production and transmission mechanisms. We also champion the need for interdisciplinary, team-led collaboration in future RA research programs.”

We have re-organized the remainder of Section 4 to outline:
A) Opportunities for investigating RA chemical composition
C) Opportunities for developing a suitable RTLF surrogate
D) Opportunities for understanding RA production mechanisms
E) Opportunities for understanding the RTLF interface

We believe that these additions and edits clarify the major themes of the Outlook.

Page 1

L34: at least in recent history, but in the 1800s it was thought that all diseases were spread through the air, miasma. Perhaps a subtle point, but worth considering that as science has evolved so too has the understanding of disease transmission.

We thank the reviewer for this comment! We acknowledge that the study of airborne disease does indeed have a long history, but it was our intention to say that aerosols are the least studied in comparison to surfaces as the route of transmission. We have edited the sentence as follows:

“Air is the main pathway for respiratory disease transmission but is also the least studied.”

L49: definitely need to add a mention here of measles.

Done! And a good point given the recent outbreaks in the US.

Page 6

Lines 13-14: Do you have a citation for the statement in lines 13-14? About chemical disinfection by atmospheric gases.

Yes, we have added a citation to the following article by Ahlawat *et al.*:

Ahlawat, A.; Mishra, S. K.; Herrmann, H.; Rajeev, P.; Gupta, T.; Goel, V.; Sun, Y.; Wiedensohler, A. Impact of Chemical Properties of Human Respiratory Droplets and Aerosol Particles on Airborne Viruses’ Viability and Indoor Transmission. *Viruses* **2022**, *14* (7), 1497. https://doi.org/10.3390/v14071497.

Additional Questions:

Quality of experimental data, technical rigor: High

Significance to chemistry researchers in this and related fields: Moderate

Broad interest to other researchers: High

Novelty: Top 1%

Is this research study suitable for media coverage or a First Reactions (a News & Views piece in the journal)?: No
